# Herpes Zoster and Subsequent Risk of Cancer: A Population-Based Study

**DOI:** 10.2188/jea.JE20120155

**Published:** 2013-05-05

**Authors:** Hui-Fen Chiu, Brian K. Chen, Chun-Yuh Yang

**Affiliations:** 1 Department of Pharmacology, College of Medicine, Kaohsiung Medical University, Kaohsiung, Taiwan; 2 Department of Health Services Policy and Management, Arnold School of Public Health, University of South Carolina, Columbia, SC, USA; 3 Department of Public Health, College of Health Sciences, Kaohsiung Medical University, Kaohsiung, Taiwan; 4 Division of Environmental Health and Occupational Medicine, National Health Research Institute, Miaoli, Taiwan

**Keywords:** herpes zoster, cancer, risk, incidence, cohort study

## Abstract

**Background:**

In this cohort study, we investigated whether a diagnosis of herpes zoster (HZ) was associated with a higher risk of subsequent cancer as compared with the Taiwanese general population.

**Methods:**

Data were obtained from the Taiwan National Health Insurance Research Database. In total, 38 743 patients who were aged 50 years or older and had received ambulatory care for HZ between 1997 and 2006 were identified as the study cohort; 116 229 age- and sex-matched patients without HZ were included as the comparison cohort. We used Cox proportional hazards regression models to estimate the hazard ratios (HRs) for subsequent cancer, after controlling for potential confounders.

**Results:**

The HR for subsequent cancer varied according to time since HZ diagnosis. The HR was 1.58 (95% CI, 1.38–1.80) within the first year, 1.30 (95% CI, 1.15–1.46) between 1 and 2 years, 1.10 (95% CI, 0.98–1.24) between 2 and 3 years, 1.02 (95% CI, 0.91–1.15) between 3 and 4 years, and 1.08 (95% CI, 0.96–1.21) between 4 and 5 years. The risk of subsequent cancer, particularly lung cancer, was significantly higher during the first 2 years after initial diagnosis of HZ.

**Conclusions:**

Our findings suggest that an HZ diagnosis is a marker of occult malignancy, particularly in lung cancer. The HRs for cancer decreased gradually over time and were no longer significant after 2 years of follow-up, which indicates that the association between HZ and cancer is likely due to detection bias.

## INTRODUCTION

Herpes zoster (HZ), or shingles, is a dermatologic manifestation of varicella infection and erupts after activation of varicella-zoster virus (VZV) previously latent in the sensory ganglia and dorsal nerve roots.^1^ VZV is considered a “re-emerging” infection because of its potential for increased prevalence in elderly adults and otherwise immunocompromised or weakened patients.^2^^,^^3^

HZ was postulated to have a direct carcinogenic effect as early as 1955^4^ and has been hypothesized to be a potential predictor of subsequent cancer.^5^ Four cohort studies have investigated the relationship between HZ infection and risk of subsequent cancer, but the results were inconsistent. Three studies found no increased risk of malignant neoplasm after HZ; however, 1 of those studies comprised only 2 groups of 50 patients.^6^ Ragozzine et al detected no significant increase in cancer risk among 590 HZ patients, as compared with rates derived from local cancer registry data in Rochester, New York.^7^ In contrast, Buntinx et al reported a higher incidence of cancer among HZ patients aged 65 years or older, but only among women.^8^ However, because no significant association was found when men and women were analyzed jointly, the authors concluded that HZ was not a marker of undiagnosed malignancy. In a comparison of malignancy rates among patients hospitalized for HZ and those expected based on the Danish Cancer Registry, Sorensen et al found that the relative risk of a cancer diagnosis during the first year of follow-up was 1.3 (95% CI, 1.1–1.5). They also detected a particularly high risk for hematologic cancer in this group (relative risk, 3.4; 95% CI, 2.3–4.9).^9^

Potential reasons for the limited empirical evidence linking HZ infection with subsequent cancer are insufficient sample size and deficient study design. We performed the present study because the epidemiologic evidence linking HZ with subsequent cancer is limited and inconsistent and because there is a need for analyses of independent data from other populations (such as Taiwan). Using national population-based data, we investigated whether patients with HZ had a higher incidence of subsequent cancer, as compared with the general population, during the first year of follow-up and whether the risk changed according to time since HZ diagnosis. In addition, we attempted to identify the most common malignancies after an HZ diagnosis.

## METHODS

### Data source

The National Health Insurance (NHI) program is a mandatory universal health insurance scheme in Taiwan and was implemented on March 1, 1995. Under the NHI, approximately 98.4% of the country’s population (22.6 million) is covered for a wide range of health care services, including ambulatory and inpatient care, Chinese medicine, dental services, childbirth, physical therapy, preventive medicine, home health care, and treatment for chronic mental illness.

The data for this study were obtained from the National Health Insurance Research Database (NHIRD), which is released by the Taiwan National Health Research Institute to researchers in Taiwan. The database is a national, population-based claims database, and all personally identifiable information is encrypted for patient protection. Hundreds of researchers have published studies based on data from the NHIRD.^10^ Because personally identifiable information in the NHIRD is encrypted to protect patient privacy, this study was exempted from full review by the Institutional Review Board of Kaohsiung Medical University.

### Study cohorts

From the dataset for tertiary care centers, we identified all patients aged 50 years or older who received ambulatory care for herpes zoster (HZ; primary diagnosis code 053 of the International Classification of Diseases, Ninth revision, Clinical Modification [ICD-9-CM]) between January 1, 1997 and December 31, 2006 (*n* = 48 218). The NHI was implemented in 1995, and we were therefore unable to identify patients who had received a new diagnosis of HZ before 1997. Thus, to limit the study subjects to new HZ cases, we excluded patients who received an HZ diagnosis before 1997 (*n* = 1197 in 1996). In addition, we excluded individuals who had received a diagnosis of cancer before (*n* = 5396) or within 2 months after (*n* = 273) an HZ diagnosis.^9^ A total of 41 352 HZ patients were ultimately selected for the study cohort group.

We defined cohort entry as the date of the first outpatient visit (index ambulatory care visit) at which subjects received HZ treatment. The comparison cohort was extracted from a random sample of 1 000 000 individuals from the entire insured population in Taiwan (approximately 22.6 million). Statistical tests showed no significant difference in age, sex, or healthcare costs between the sample group and entire population, as reported by the NHRI. In constructing the comparison group, we first excluded patients younger than 50 years who had received a diagnosis of HZ between 1996 and 2010. Then, for each study cohort patient, 3 reference subjects from the remaining patients in the population-based database were selected randomly and matched by sex, age, and year of index ambulatory care visit. For the comparison cohort patients, who were selected using risk-set sampling, we assigned an index date identical to that of the index ambulatory care index visit of the matched study cohort. In addition, we excluded patients who had received a cancer diagnosis before or within 60 days of the date of cohort entry. For the 41 352 HZ patients thus identified, no controls could be found for only 2609 cases. A total of 116 229 subjects served as our comparison cohort group.

### Potential confounders

For all members in both cohorts, we acquired data on potential confounders such as degree of urbanization of the individuals’ residence community and level of insurance (as the economic index). In Taiwan, urbanization levels are divided into 8 categories, ranging from category 1 (the most urbanized) to category 8 (least urbanized).^11^ In our analyses, the urban–rural classification was aggregated into 4 levels: I, metropolitan (categories 1 and 2); II, city (categories 3 and 4); III, town (categories 5 and 6); and IV, rural (categories 7 and 8). Furthermore, we included as confounders a diagnosis of diabetes mellitus (DM)^12^ and the Charlson comorbidity index (CCI) for the 12 months before the index date.^13^

### Statistical analysis

We used the χ^2^ test to compare differences in proportions between the study and comparison cohorts. Each patient was followed from the date of cohort entry to the first date of cancer diagnosis, date of death, or the end of the study (December 31, 2010), whichever occurred first. Cancer diagnosis was defined according to the criteria of the Registry for Catastrophic Illness Patient Database, a separate subsection of the NHIRD. The registry is considered accurate because all cancer diagnoses must be validated by a histologic test before a patient is listed therein. Such rigorous proof is required because patients who receive a diagnosis of a catastrophic illness are exempt from all copayments, according to NHI policies.

Cancer incidence rates were calculated by dividing the number of cancer patients by the total person-years of follow-up. We used log-rank tests to evaluate the difference in the risk of subsequent cancer between the 2 cohorts. Furthermore, we used Cox proportional hazards regression models to estimate hazard ratios (HRs) and 95% CIs for subsequent cancer, while controlling for the above-mentioned potential confounders.^14^ To validate the proportional hazards assumptions in the Cox regression model, we used diagnostic log-log survive plots to check each dichotomous variable in the model for proportionality. These diagnostic tests revealed that the proportional assumption was satisfied. Analyses were performed using SAS (version 9.2, SAS Institute Inc., Cary, NC, USA). All statistical tests were 2-sided, and a *P* value of less than 0.05 was considered to indicate statistical significance.

## RESULTS

The 38 743 patients in our HZ cohort were compared with the 116 229 selected matched controls. Table 1 shows selected demographic characteristics and medical conditions of the study subjects. As compared with patients without HZ, patients with HZ were more likely to have diabetes, to have a higher Charlson comorbidity index and lower monthly income, and to live in more-urbanized areas of Taiwan (Table 1).

**Table 1.** Basic characteristics of patients with and without herpes zoster (HZ)

Variable	HZ patients (*n* = 38 743)		Comparison patients (*n* = 116 229)		*P* value
	
	*n*	%	*n*	%	
Sex					
Male	20 514	52.95	61 542	52.95	
Female	18 229	47.05	54 687	47.05	
Age, years					
50–59	12 174	31.42	36 679	31.56	0.8824
60–69	11 847	30.58	35 490	30.53	
≥70	14 722	38.00	44 060	37.91	
Diabetes					
Yes	4984	12.86	11 308	9.73	<0.0001
No	33 759	87.14	104 921	90.27	
Monthly income (NT$)					
Financially dependent	11 942	30.82	33 855	29.13	<0.0001
1–15 840	10 066	25.98	22 550	19.40	
15 841–25 200	9818	25.34	43 673	37.57	
≥25 201	6917	17.85	16 151	13.90	
Urbanization					
Metropolitan	24 068	62.12	48 105	41.39	<0.0001
City	6509	16.80	28 048	24.13	
Town	5528	14.27	25 957	22.33	
Rural	2638	6.81	14 119	12.15	
Charlson comorbidity index (means ± SD)	0.41 ± 0.84		0.29 ± 0.67		<0.0001

Among the 154 972 sampled patients, 1065 (0.69%) developed cancer during the first year of follow-up: 376 (0.97% of HZ patients) from the study cohort (incidence rate, 9.74 per 1000 person-years) and 689 (0.59%) from the comparison cohort (incidence rate, 5.94 per 1000 person-years).

Patients with HZ were more likely than patients in the comparison cohort to develop cancer during the first year of follow-up (crude HR, 1.65; 95% CI, 1.45–1.87), and this increase in risk remained significant after adjustment (HR, 1.58; 95% CI, 1.38–1.80). After age stratification, the adjusted HR for subsequent cancer was 1.58 (95% CI, 1.12–2.21) in those aged 50 to 59 years, 1.45 (95% CI, 1.11–1.90) in those aged 60 to 69 years, and 1.68 (95% CI, 1.45–2.01) in those aged 70 years or older. In sex-stratified analyses, the HR for subsequent cancer was slightly higher for female HZ patients (HR, 1.61; 95% CI, 1.30–2.00) than for male HZ patients (HR, 1.55; 95% CI, 1.31–1.84). However, the difference was not significant (Table 2). The log-rank test showed that the 1-year cancer-free survival rate was significantly lower in patients with HZ than in the comparison cohort (*P* < 0.001).

**Table 2.** Hazard ratios (HRs) for cancer during the first-year of follow-up among patients with and without herpes zoster (HZ), stratified by age and sex

	No. of cancers	Follow-up in person-years	Incidence (per 1000 person-years)	Crude HR (95% CI)	Adjusted HR (95% CI)
All					
Comparison	689	115 952.04	5.94	1.00	1.00
HZ	376	38 587.08	9.74	1.65 (1.45–1.87)	1.58 (1.38–1.80)^a^
Age, years					
50–59					
Comparison	107	36 631.42	2.92	1.00	1.00
HZ	59	12 153.32	4.85	1.68 (1.22–2.31)	1.58 (1.12–2.21)^b^
60–69					
Comparison	197	35 409.59	5.56	1.00	1.00
HZ	92	11 807.09	7.79	1.44 (1.12–1.85)	1.45 (1.11–1.90)^b^
≥70					
Comparison	385	43 911.03	8.77	1.00	1.00
HZ	225	14 626.68	15.38	1.75 (1.48–2.07)	1.68 (1.41–2.01)^b^
Sex					
Female					
Comparison	252	54 586.87	4.62	1.00	1.00
HZ	142	18 170.10	7.82	1.69 (1.38–2.08)	1.61 (1.30–2.00)^c^
Male					
Comparison	437	61 365.18	7.12	1.00	1.00
HZ	234	20 416.98	11.46	1.62 (1.38–1.90)	1.55 (1.31–1.84)^c^

^a^Adjusted for age, sex, diabetes status, monthly income, Charlson comorbidity index, and urbanization level.

^b^Adjusted for sex, diabetes status, monthly income, Charlson comorbidity index, and urbanization level.

^c^Adjusted for age, diabetes status, monthly income, Charlson comorbidity index, and urbanization level.

Table 3 shows the HRs for subsequent cancer according to time to malignancy after the index ambulatory care visit. The HR was 1.58 (95% CI, 1.38–1.80) within the first year, 1.30 (95% CI, 1.15–1.46) between 1 and 2 years, 1.10 (95% CI, 0.98–1.24) between 2 and 3 years, 1.02 (95% CI, 0.91–1.15) between 3 and 4 years, and 1.08 (95% CI, 0.96–1.21) between 4 and 5 years. The observed association increased when we included those who received a cancer diagnosis within 2 months after an HZ diagnosis. The HR for subsequent cancer was 2.18 (95% CI, 1.93–2.46) within the first year. A significant increase in the risk of subsequent cancer was observed only during the first 2 years after an initial diagnosis of HZ.

**Table 3.** Hazard ratios (HRs) for cancer after a herpes zoster (HZ) diagnosis, stratified by time to cancer diagnosis

Time interval	No. of cancers	Follow-up in person-years	Incidence (per 1000 person-years)	Crude HR (95% CI)	Adjusted HR^a^ (95% CI)
1st year					
Comparison	689	115 952.04	5.94	1.00	1.00
HZ	376	38 587.08	9.74	1.65 (1.45–1.87)	1.58 (1.38–1.80)
Between 1 and 2 years					
Comparison	955	230 595.69	4.14	1.00	1.00
HZ	411	76 519.36	5.37	1.31 (1.17–1.47)	1.30 (1.15–1.46)
Between 2 and 3 years					
Comparison	1087	343 196.58	3.17	1.00	1.00
HZ	407	113 653.53	3.58	1.13 (1.01–1.26)	1.10 (0.98–1.24)
Between 3 and 4 years					
Comparison	1170	453 400.81	2.58	1.00	1.00
HZ	393	149 964.79	2.62	1.02 (0.91–1.14)	1.02 (0.91–1.15)
Between 4 and 5 years					
Comparison	1185	560 973.74	2.11	1.00	1.00
HZ	420	185 545.09	2.26	1.08 (0.97–1.21)	1.08 (0.96–1.21)

^a^Adjusted for age, sex, diabetes status, monthly income, Charlson comorbidity index, and urbanization level.

Table 4 shows HRs for the most commonly observed sites of malignancy within the first year. A significantly increased risk was noted for lung cancer among women and lung and prostate cancers among men. When data on cancer development during the first 2 years were analyzed, the most commonly observed sites of malignancy were the same as those seen during the first year, and the HRs for the respective cancer sites were similar for the different intervals (data not shown).

**Table 4.** Hazard ratios (HRs) for cancer after a herpes zoster diagnosis, stratified by site of cancer

Cancer site	Women			Men		
	
	No. of cases/ No. of controls	Crude HR (95% CI)	Adjusted HR^a^ (95% CI)	No. of cases/ No. of controls	Crude HR (95% CI)	Adjusted HR^a^ (95% CI)
Colon/rectum	11/45	0.73 (0.38–1.42)	0.67 (0.33–1.35)	29/74	1.20 (0.78–1.85)	1.12 (0.70–1.77)
Lung	21/28	2.33 (1.32–4.13)	2.35 (1.27–4.33)	40/60	2.00 (1.34–2.98)	2.12 (1.36–3.30)
Liver	19/26	2.15 (1.19–3.89)	1.97 (0.98–3.97)	28/68	1.24 (0.80–1.92)	1.25 (0.75–2.07)
Breast	18/41	1.32 (0.76–2.29)	1.21 (0.65–2.22)			
Cervix uteri	9/18	1.50 (0.67–3.34)	1.58 (0.60–4.13)			
Prostate				33/52	1.97 (1.27–3.06)	1.90 (1.18–3.09)
Gastric				23/35	1.96 (1.16–3.31)	1.50 (0.80–2.79)

^a^Adjusted for age, diabetes status, monthly income, Charlson comorbidity index, and urbanization level.

## DISCUSSION

To our knowledge, the present cohort is the largest (38 743 patients with HZ) to be studied in order to estimate the risk of subsequent malignancy after an HZ diagnosis. In this population-based cohort study, we found that HZ patients were 1.58 times as likely as the general population to develop subsequent cancer during a 1-year follow-up period, after controlling for potential confounders.

It is important to note that our study may suffer from detection bias, as would any study that attempts to investigate increased incidence of a disease (cancer) in relation to a clinical diagnosis of another disease (HZ).^8^ We therefore intentionally biased our results downward by excluding patients who received a diagnosis of cancer during the first 2 months after an HZ diagnosis. Indeed, the HR for subsequent cancer within the first year was higher when we included those diagnosed with cancer within the first 2 months after an HZ diagnosis. Increased diagnostic testing after an HZ diagnosis could potentially explain the association between HZ and cancer in the short term. We found that HRs decreased gradually over time, that they were significant only for the first 2 years of follow-up, and that the HR was highest for the first year. These findings are consistent with the conclusion that the association between HZ and cancer is due to detection bias.

An increased risk of cancer long after an HZ diagnosis would suggest that the active infection was directly carcinogenic or that cancer risk was increased due to an underlying immune deficiency.^7^ In the present study, the relative risk of subsequent cancer varied according to duration of follow-up after HZ diagnosis. The HR for cancer showed a gradual temporal decrease, was statistically significant only during the first 2 years of follow-up, and was highest during the first year after diagnosis of HZ. These results suggest that HZ is not likely to be a risk factor for cancer. Rather, the increased risk of subsequent cancer during the 2 years after an HZ diagnosis may reflect the presence of occult cancer shortly after HZ diagnosis.^7^ Our results support the hypothesis that HZ is an indicator of occult malignancy. This hypothesis was supported in 1 previous study^9^ and contradicted in 3 other studies, which reported no association.^6^^–^^8^ However, only 113 cancer cases were investigated in those 3 studies, so it is highly unlikely that the studies had sufficient statistical power to detect an effect for rare events such as cancer or to assess site-specific cancer risks.

If an HZ diagnosis is a marker of occult malignancy, we would expect that both men and women would be at increased risk for a site-specific cancer. Only 2 studies conducted a sex-stratified analysis of the risks of site-specific cancer after HZ. Ragozzino et al found no increase in the relative risks of cancer at specific sites, except for colon and bladder cancers in women.^7^ Likewise, another study reported a borderline statistically significant effect for colorectal cancer among women only.^8^ The absence of statistically significant associations in men may indicate either insufficient statistical power or a weak and/or absent relationship between HZ and cancer in men.^8^ To our knowledge, our study is the first to find that both male and female HZ patients were more likely than the general population to develop subsequent lung cancer. Our finding of an increased risk of subsequent prostate cancer after HZ is also particularly noteworthy given that no other studies have examined the association between HZ and prostate cancer. Nevertheless, the possibility that ours is a chance finding should be considered, because existing evidence for an association between the risks of cancer at specific sites and HZ is inconsistent.

The mechanisms that might explain the increased risk of subsequent malignancy in patients with HZ are unclear. Before diagnosis, cancers are often present in a preclinical or undetectable form. Thus, HZ could be an early manifestation of immune impairment associated with malignancy.^8^ An alternative hypothesis is that reactivation of VZV triggers immunologic mechanisms, such as tissue antigen alteration or antigenic stimulation, which could result in cancer.^8^ Reports that malignancies had developed at the sites of previous HZ infection are consistent with this hypothesis.^4^^,^^15^^,^^16^ Finally, cell-mediated immunity has an important role in reactivation of VZV^17^^,^^18^ and maintenance of immunologic surveillance control of malignancy.^19^^,^^20^ A disorder in host immunity could lead to both HZ and malignancy, either of which may appear first.^21^

This study has a number of strengths, including a well-defined source population, use of an external comparison group selected from the general population, and a large sample size. Nevertheless, we note several limitations of the present study. First, miscoding and misclassification are potential biases in an established database that relies on physician-reported diagnoses. However, such miscoding and misclassification are highly unlikely to be differential (ie, it is unlikely that miscoding would differ between patients with and without HZ) and would therefore tend to underestimate rather than overestimate the true association. Second, because the NHIRD database masks patient identities, we were unable to use histologic reports to verify diagnoses. However, because enrollment in the catastrophic illness registry is based on evidence from pathology and/or cytology, the data on cancer occurrence should be accurate. Third, our study may suffer from data censorship because some patients with HZ may not have visited a doctor. However, access to healthcare is excellent in Taiwan because the NHI provides universal coverage with very low financial barriers and has no gatekeeping role that might prevent access to specialized care. During the study period, patients were responsible for a copayment of only US$3 to $15 for each visit. Given the minimal barriers to access in Taiwan, we believe that most patients with a painful disease such as HZ would seek medical attention after disease onset. The number of HZ cases not captured by Taiwan’s national health care database is likely to be small.^22^ Fourth, although we controlled for several potential confounders in our statistical analysis, due to data limitations we were not able to include a number of possible cancer-related confounding variables such as smoking history, dietary habits, and alcohol use. Fifth, we limited our case cohort to patients at tertiary care centers. Selection biases may therefore exist, and caution must be used when interpreting the results. Sixth, more than 98% of Taiwan’s population is of Han Chinese ethnicity, so the results of the present study may not be generalizable to other ethnic groups. Seventh, a spurious association between HZ and risk of subsequent malignancy could occur if patients with HZ were more likely to be screened for cancer. However, such surveillance bias is minimal because there is limited public attention highlighting HZ as a risk factor for cancer. As a result, HZ infection seldom prompts physicians to heighten alertness to cancer risk or increase clinical surveillance. Finally, it is possible that we have not accounted for residual unmeasured confounding factors that differentially affect the case and comparison cohorts.

In summary, we found a higher risk of subsequent cancer, particularly lung cancer, in the first 2 years of follow-up after a diagnosis of HZ. Our findings suggest that an HZ diagnosis is a marker of occult malignancy. After HZ diagnosis, cancer HRs decreased gradually over time and were no longer significant after 2 years of follow-up; the HR was highest during the first year. Our findings also suggest that the association between HZ and cancer resulted from detection bias. Only 0.97% of patients with HZ received a cancer diagnosis within the first year of follow up, and only 0.16% of HZ patients received a subsequent diagnosis of lung cancer. Thus, cancer screening for patients with HZ would have limited value and is unlikely to be cost-effective.^9^

## References

[1] Jih JS, Chen YJ, Lin MW, Chen YC, Chen TJ, Huang YL, Epidemiological features and costs of herpes zoster in Taiwan: a national study 2000 to 2006. Acta Derm Venereol. 2009;89:612–6 10.2340/00015555-0729 19997693

[2] Gilden DH, Cohrs RJ, Mahalingam R Clinical and molecular pathogenesis of varicella virus infection. Viral Immunol. 2003;16:243–58 10.1089/088282403322396073 14583142

[3] LaGuardia JJ, Gilden DH Varicella-zoster virus: a re-emerging infection. J Investig Dermatol Symp Proc. 2001;6:183–7 10.1046/j.0022-202x.2001.00041.x 11924825

[4] Wyburn-Mason R Malignant change arising in tissues affected by herpes. Br Med J. 1955;2:1106–9 10.1136/bmj.2.4948.1106 13260671 PMC1981341

[5] Smith JB, Fenske NA Herpes zoster and internal malignancy. South Med J. 1995;88:1089–92 10.1097/00007611-199511000-00001 7481976

[6] Fueyo MA, Lookingbill DP Herpes zoster and occult malignancy. J Am Acad Dermatol. 1984;11:480–2 10.1016/S0190-9622(84)70195-2 6480952

[7] Ragozzino MW, Melton LJ 3rd, Kurland LT, Chu CP, Perry HO Risk of cancer after herpes zoster: A population-based study. N Engl J Med. 1982;307:393–7 10.1056/NEJM198208123070701 6979711

[8] Buntinx F, Wachana R, Bartholomeeusen S, Sweldens K, Geys H Is herpes zoster a marker for occult or subsequent malignancy? Br J Gen Pract. 2005;55:102–7 15720930 PMC1463183

[9] Sørensen HT, Olsen JH, Jepsen P, Johnsen SP, Schønheyder HC, Mellemkjaer L The risk and prognosis of cancer after hospitalization for herpes zoster: A population-based follow-up study. Br J Cancer. 2004;91:1275–9 10.1038/sj.bjc.6602120 15328522 PMC2409892

[10] Chen YC, Yeh HY Taiwan’s National Health Insurance Database: administrative health care database as study object in biblometrics. Scientometrics. 2011;86:365–80 10.1007/s11192-010-0289-2

[11] Tzeng GH, Wu TY Characteristics of urbanization levels in Taiwan districts. Geographic Res. 1986;12:287–323

[12] Giovannucci E, Harlan DM, Archer MC, Bergenstal RM, Gapstur SM, Habel LA, Diabetes and cancer: a consensus report. Diabetes Care. 2010;33:1674–85 10.2337/dc10-0666 20587728 PMC2890380

[13] Deyo RA, Cherkin DC, Ciol MA Adapting a clinical comorbidity index for use with ICD-9-CM administrative databases. J Clin Epidemiol. 1992;45:613–9 10.1016/0895-4356(92)90133-8 1607900

[14] Hosmer DW, Lemeshow S. Applied survival analysis. New York: John Wiley & Sons, Inc; 1999. p. 317–26.

[15] Rogers RS 3rd, Tindall JP Geriatric herpes zoster. J Am Geriatr Soc. 1971;19:495–504 5094651 10.1111/j.1532-5415.1971.tb01208.x

[16] Wolff HH, Wendt V, Winzer M Cutaneous pseudolymphoma at the site of prior herpes zoster eruption. Arch Dermatol Res. 1987;279 Suppl:S52–4 10.1007/BF00585920 2959212

[17] Arvin A Aging, immunity, and the varicella-zoster virus. N Engl J Med. 2005;352:2266–7 10.1056/NEJMp058091 15930416

[18] Weller TH Varicella and herpes zoster. Changing concepts of the natural history, control, and importance of a not-so-benign virus. N Engl J Med. 1983;309:1434–40 10.1056/NEJM198312083092306 6195526

[19] Dunn GP, Old LJ, Schreiber RD The immunobiology of cancer immunosurveillance and immunoediting. Immunity. 2004;21:137–48 10.1016/j.immuni.2004.07.017 15308095

[20] Konjević G, Jurisić V, Banićevic B, Spuzić I The difference in NK-cell activity between patients with non-Hodgkin’s lymphomas and Hodgkin’s disease. Br J Haematol. 1999;104:144–51 10.1046/j.1365-2141.1999.01129.x 10027727

[21] Ho JD, Xirasagar S, Lin HC Increased risk of a cancer diagnosis after herpes zoster ophthalmicus: A nationwide population-based study. Ophthalmology. 2011;118:1076–81 10.1016/j.ophtha.2010.10.008 21232800

[22] Lin HC, Chien CW, Ho JD Herpes zoster ophthalmicus and the risk of stroke: A population-based follow-up study. Neurology. 2010;74:792–7 10.1212/WNL.0b013e3181d31e5c 20200348

